# Vaginal Sheets with *Thymbra capitata* Essential Oil for the Treatment of Bacterial Vaginosis: Design, Characterization and *In Vitro* Evaluation of Efficacy and Safety

**DOI:** 10.3390/gels9040293

**Published:** 2023-04-02

**Authors:** Mariana Tomás, Lúcia G. V. Sousa, Ana Sofia Oliveira, Carolina P. Gomes, Ana Palmeira-de-Oliveira, Carlos Cavaleiro, Lígia Salgueiro, Nuno Cerca, José Martinez-de-Oliveira, Rita Palmeira-de-Oliveira

**Affiliations:** 1 CICS-UBI, Health Sciences Research Center, Faculty of Health Sciences, University of Beira Interior, 6201-506 Covilhã, Portugal; 2 Laboratory of Research in Biofilms Rosário Oliveira (LIBRO), Centre of Biological Engineering (CEB), University of Minho, 4710-057 Braga, Portugal; 3 LABBELS—Associate Laboratory, 4710-057 Braga, Portugal; 4 Labfit-HPRD Health Products Research and Development, Lda Edifício UBIMedical, Estrada Municipal 506, 6200-281 Covilhã, Portugal; 5 CIEPQPF, Chemical Process Engineering and Forest Products Research Centre, University of Coimbra, 3030-790 Coimbra, Portugal; 6 Faculty of Pharmacy, University of Coimbra, 3000-548 Coimbra, Portugal

**Keywords:** vaginal infections, bacterial vaginosis, treatment, vaginal sheet, *Thymbra capitata* essential oil, *Gardnerella* several species (spp.)

## Abstract

We aimed to incorporate *Thymbra capitata* essential oil (TCEO), a potent antimicrobial natural product against bacterial vaginosis (BV)-related bacteria, in a suitable drug delivery system. We used vaginal sheets as dosage form to promote immediate relief of the typical abundant vaginal discharge with unpleasant odour. Excipients were selected to promote the healthy vaginal environment reestablishment and bioadhesion of formulations, while the TCEO acts directly on BV pathogens. We characterized vaginal sheets with TCEO in regard to technological characterization, predictable in vivo performance, in vitro efficacy and safety. Vaginal sheet D.O (acid lactic buffer, gelatine, glycerine, chitosan coated with TCEO 1% *w/w*) presented a higher buffer capacity and ability to absorb vaginal fluid simulant (VFS) among all vaginal sheets with EO, showing one of the most promising bioadhesive profiles, an excellent flexibility and structure that allow it to be easily rolled for application. Vaginal sheet D.O with 0.32 µL/mL TCEO was able to significantly reduce the bacterial load of all in vitro tested *Gardnerella* species. Although vaginal sheet D.O presented toxicity at some concentrations, this product was developed for a short time period of treatment, so this toxicity can probably be limited or even reversed when the treatment ends.

## 1. Introduction

Bacterial vaginosis (BV) is caused by a replacement of saprophytic flora by anaerobe bacteria which act together through synergic mechanisms to form dense and structured biofilms. Polymicrobial biofilms are mainly constituted by *G. vaginalis* clusters, that strongly adhere to the vaginal epithelium, and are associated with other bacteria such as *Fannyhessea vaginae*, *Mobiluncus* spp. and *Prevotella* spp. [1,2,3,4,5,6,7]. Indeed, the interactions between *G. vaginalis* and other BV-associated bacteria are extremely complex and may involve the modulation of virulence factors and other biological processes which allow an intimate cooperation and exceptional organization that represents a challenge for treatment [8,9,10,11]. Although *Gardnerella* species have been detected as the most common bacteria present in cases of BV, the role of this pathogen in BV is still controversial, since *Gardnerella* colonization does not always cause clinical episodes of infection [12]. Recent studies also changed the state of the art of this topic by showing that *G. vaginalis*, as previously referred, actually includes 13 different species of the genus *Gardnerella*, of which 4 have been described: *G. vaginalis*, *G. piotii*, *G. leopoldii*, and *G. swidsinskii* [13]. Currently, it is not totally clear the role of each species of *Gardnerella* in the initialization of infection and their respective virulence potential.

Alternative treatments for BV have been studied in view of reducing recurrences after standard antimicrobial treatments [6,14]. Natural compounds with antimicrobial activity and substances that aim to re-establish natural saprophytic flora (probiotics, prebiotics and acidifying agents) have been proposed as alternative strategies to treat BV [14]. Traditionally, plants have been used for the treatment of vaginal infections. Nowadays, natural compounds (namely essential oils) tend to be valued by some women, and may even improve the acceptability among women that are more prone to use natural products [14]. The potential of *Artemisia princeps Pamp*., thyme and *Thymbra capitata* essential oils against BV-related bacteria has been reported [14]. *Artemisia princeps Pamp*. essential oil (particularly α-terpineol) can be an alternative treatment of BV, since it inhibited the growth of *G. vaginalis* (MIC value of 0.06% (*v/v*) and also inhibited the expressions of proinflammatory cytokines and the activation of NF-κB and increased expression of the anti-inflammatory cytokine IL-10 in a study involving mice [15]

Furthermore, thyme essential oil and its component thymol (a small hydrophobic molecule able to interact with the lipid bilayer of membranes, promoting the loss of integrity and the leakage of cellular material) also demonstrated an inhibitory effect on newly formed biofilms (acting in the initial stages of biofilm organization, namely in the attachment to the substrate) and mature *G. vaginalis* biofilms in vitro [16,17]

TCEO exhibited potent antibacterial activity against *G. vaginalis* planktonic cells (MIC and MLC values of 0.16 µL/mL). Additionally, TCEO presented significant antibiofilm activity (in the range 0.16 to 0.64 µL/mL). This activity was higher than its main compound (carvacrol), suggesting this effect was not only dependent on carvacrol but also on a synergic action of major and minor components of the oil. This antimicrobial effect was attributed to the hydrophobicity that causes damage to the cellular membrane leading to cell destruction. Importantly, TCEO presented a selective antibacterial action, since it was significantly less effective against lactobacilli, with MIC (1.25–2.50 μL/mL) and MLC values (1.25–2.50 μL/mL) almost tenfold higher than the concentrations needed for *G. vaginalis* [1]. Similarly, recent results from our research group showed the potent antimicrobial activity of two TCEOs against a clinical isolate of *Gardnerella* sp. recovered from cases of BV (MIC 0.04 and 0.08 µL/mL; MLC 0.08 and 0.16 µL/mL). Both TCEOs, at 0.16 µL/mL, caused a biofilm mass reduction of 20–30% on a 48 h biofilm of *Gardnerella* sp. UM241. TCEO (batch collected in Carvoeiro, Portugal) at 0.16 µL/mL exhibited a biomass reduction of 20–30% in *G. vaginalis* UM137, *G. piotii* UM035 and *G. swidsinskii* GS 9838-1, being less effective against *G. leopoldii* UGent 09.48 (40% biomass reduction) [18]. Although recent advances in the genetic classification of *Gardnerella* spp. brought changes to the classification of bacteria formerly known as *G. vaginalis*, these results support the overall interest of this essential oil for bacteria involved in BV in concentrations up to 0.64 µL/mL [13]. Recently, a study demonstrated that TCEO also presented antimicrobial effect against polymicrobial biofilms formed by *G. vaginalis*, *F. vaginae, Lactobacillus iners, Mobiluncus curtisii*, *Peptostreptococcus anaerobius* and *P. bivia* [19].

In this study we aimed to incorporate this potent active substance in a suitable vaginal drug delivery system that may simultaneously control symptoms and optimize TCEO efficacy, while preventing its volatilization. We aimed to develop and characterize vaginal sheets with TCEO in regard to technological characterization, predictable in vivo performance, in vitro efficacy against *Gardnerella* spp. and safety. In previous studies, our research group has proposed a formulation approach specifically designed for BV that absorbs the excessive amount of vaginal fluid that characterizes this pathology as a swelling-hygroscopic formulation, immediately contributing to the relief of one of the symptoms that causes discomfort [20]. The vaginal sheet is a variation of vaginal films but unlike vaginal films it is not designed to dissolve immediately for drug release. In particular, this formulation is bigger and thicker than films and must present the ability to absorb vaginal fluids without losing its essential structure in a short time [20].

A rational design approach was followed to achieve the purpose of the study. Excipients were selected based on their safety for vaginal application and ability to fulfil the goals of the dosage form: gelatine was used as the main macromolecule in all formulations, and bioadhesive polymers such as PVA, HPMC and chitosan were selected to promote the efficient contact with the epithelium to avoid leakage [21,22,23,24,25,26,27,28,29,30,31,32,33,34,35,36,37,38]. Furthermore, chitosan can contribute to the disruption of BV-related biofilms, and also improve the treatment with metronidazole when used as an excipient to formulate liposomes [14,39,40,41,42,43,44,45,46].

The pH buffer solution (acid lactic/sodium lactate) was elected to promote restoration of vaginal pH upon administration [21,22,31,32]. Some studies suggest that lactic acid, used as acidifying agent, can normalize vaginal dysbiosis, promoting lactobacilli colonization and simultaneously inhibiting the growth of pathogenic bacteria [14,47,48,49]. Additionally, powdered active substances with hygroscopic properties were included in the vaginal sheet.

Glycerine and propylene glycol were selected as plasticizers. Both are commonly used in vaginal products, including films, and are considered safe, although at high concentrations they can confer high osmolality to formulations which leads to toxicity [22,50,51,52,53,54,55]. Propylene glycol showed a higher potential to cause toxicity [56]. Therefore, glycerine was preferentially used as the plasticizer.

## 2. Results and Discussion

### 2.1. Freeze-Drying Efficiency

The solvent casting method is the process most often reported in the literature for the preparation of vaginal films, although the freeze-drying process has also been explored for films, namely for wound healing [38,57,58].

The removal of water from formulations was achieved (freeze-drying efficiency 93.8% ± 3.0 (D) to 98.5% ± 0.5 (A)) (Supplementary Materials S1). Low water content is desirable in solid formulations to prevent microbial contamination.

### 2.2. Sensorial Characteristics

Sensorial characteristics of vaginal sheets are presented in Table 1 and photographs are presented in Supplementary Materials S2.

The physical characteristics and sensorial perception of vaginal products influence the acceptability and the adherence of users [59]. In general, women prefer vaginal products to be colourless (or light-coloured) and odourless. Natural ingredients tend to be valued by many people [59,60,61].

Previous studies reported the acceptability of vaginal films for the prevention of HIV transmission. Fan et al. reported that women most frequently preferred vaginal films to be smooth and thin, translucent and square shaped (5.08 *×* 5.08 cm). Most women preferred odourless, colourless and flavourless films, to keep the natural characteristics of vaginal fluids. Women also preferred translucent or opaque films, since transparent films are associated with a minimum level of visual discernibility [60]. Guthrie et al. reported that 33.3% of women within their study preferred films sized as 7.62 *×* 2.54 cm. Additionally, women related the size with easiness of insertion, efficacy and leakage. Films with texture were expected to improve insertion by providing better grip on the film with 50% of participants preferring the film with one smooth side and one textured side for this reason [61].

In our study, the final size of the vaginal sheets (7 *×* 2.4 cm) was defined considering the mean dimensions of the human vagina to allow a complete coverage and the reported data from literature. The light-coloured vaginal sheets obtained were not expected to promote significant changes to vaginal fluids. The odour of the TCEO vaginal sheets is typical from the natural extract and was expected to stand as an advantage for the product by masking the characteristic unpleasant odour of BV vaginal fluid. Furthermore, the fact that TCEO is a natural active substance may even improve acceptability among women who are more prone to use natural products/ingredients [62].

### 2.3. Thickness

The thickness of vaginal sheets ranged from 0.813 ± 0.046 mm (base formulation D) to 1.111 ± 0.176 mm (base formulation B)—Table 2.

This variation between formulations can be explained by differences in composition, particularly related to the polymers used and their concentrations. Vaginal sheets are thicker than vaginal films, since the freeze-drying process allows them to maintain the porous structure originating from the network structure of gels [20]. It was observed that these vaginal sheets presented homogeneous thicknesses. The addition of TCEO to the base formulations did not change the structure of the vaginal sheets (thickness was similar).

### 2.4. pH and Buffer Capacity

After the dissolution of vaginal sheets in VFS pH 5 (1:10 *w/w*), the resultant pH was closer to the healthy vaginal fluid pH (Table 2), suggesting that vaginal sheets with the LA buffer solution can contribute to the correction of the VFS pH and contribute to reestablishment of healthy vaginal conditions.

**Figure 1:**
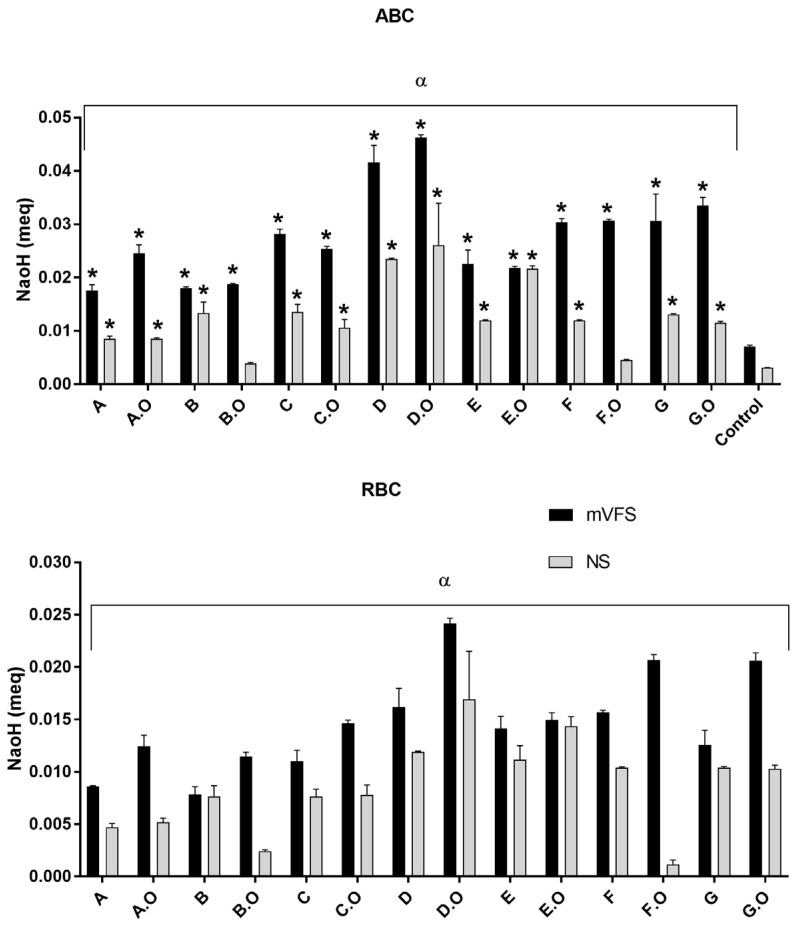
Determination of Absolute Buffer Capacity (part 1) and Relevant Buffer Capacity (part 2) of base formulations and sheets with TCEO. Bars represent the mean of 3 determinations and lines the standard deviation. NS = normal saline; mVFS = vaginal fluid simulant. * represents statistically significant difference from control (*p* < 0.05); α represent that the mean value for each formulation is different compared to the solvent (mVFS or saline solution).

The addition of TCEO to the base formulations caused a slight decrease in pH (significantly different for all formulations, *p* < 0.05, Tukey’s Multiple Comparison Test). We studied the buffer capacity of the formulations (Figure 1).

We included two solvents for the dilution of vaginal sheets: mVFS and saline solution (since it has a lower buffer capacity) [20,52,53]. The mean value of RBC and ABC for each vaginal sheet considering the solvent (mVFS or saline solution) was significantly different (two-way ANOVA, *p* < 0.05), highlighting some contributions from the solvent to the final buffer capacity.

The ABCs of all vaginal sheets were significantly different from controls except for B.O and F.O (Dunnett’s multiple comparisons test, *p* < 0.05). Thus, the acid-buffer capacity was attributed to the vaginal sheets, although there were small contributions of the solvents in which they were dissolved.

D.O exhibited the higher ABC and RBC, which is in line with expectations since this formulation contains the highest strength buffer. This ability to reduce the vaginal pH represents a therapeutic advantage for the treatment of BV [48,63,64,65,66]. Indeed, topically applied lactic acid and acidic vehicles such as gels, have been proposed as treatments for BV.

### 2.5. Absorption Efficiency of mVFS

This dosage form is particularly interesting for the treatment of BV, since it can contributes to the relief of one of the most important symptom which is associated with a significant negative impact on women’s daily life [20]. Comparing with gels, vaginal sheets (similarly to films) avoid leakage and messiness [20,25,38]. Initially, vaginal sheets presented a swelling behaviour, absorbing the mVFS (with pH and volume adapted to the pathological condition). After that, and depending on their composition, vaginal sheets begin to form a viscous gel, resulting from the dissolution of some components into the mVFS, that is, they begin to lose their structure. As for films, the rate of dissolution/dispersion of vaginal sheets is related to the choice of the type of polymer (hydrophilic films experience rapid dissolution in the presence of aqueous solvents such as vaginal fluid, while hydrophobic polymers can be useful to extend the residence time and the drug release time) [38]. For the prepared vaginal sheets, the hydrophilic polymers did not immediately dissolve in the presence of VFS. Instead, a first swelling step occurred as a result of the hygroscopic properties of the gelatine, resulting in a lower dissolution rate compared to vaginal films.

Formulation E.O presented the lowest absorption efficiency of mVFS (47% ± 2 after 30 min) and the entire sheet was dissolved in mVFS, with total loss of the initial structure and formation of a gel after 7 h. This formulation contains less gelatine compared to the other formulations, which makes the gelling process quicker and allows the polymers to dissolve earlier. Furthermore, this formulation contains PVA, which is a fast-dissolving polymer, commonly used to formulate rapidly dissolving vaginal films such as for anti-HIV products [23,38,67,68].

Among the base formulations, formulation B presented the highest absorption ability (84% ± 14 after 11 h) (Table 3). Among sheets with TCEO, D.O exhibited the highest absorption ability (63% ± 0 after 7 h). Vaginal sheet D.O exhibited a typical swelling behaviour until 7 h. After this timepoint, it began to dissolve in mVFS and gradually formed a gel. The absorption profile of vaginal sheet D.O can be particularly relevant in the context of a possible clinical use if, similarly to almost all vaginal products, it is intended to be applied at bedtime. This would allow the vaginal sheet to absorb the excess of vaginal fluid during the night period and gradually becoming a bioadhesive gel during the daytime (avoiding leakage). It was observed that even after 24 h, vaginal sheet D.O did not totally lose its structure. This formulation contained one of the highest concentrations of gelatine and it also contained 1.5% *w/w* chitosan, which explains its high affinity to absorb aqueous medium.

### 2.6. Textural Analysis: Hardness and Resilience

**Figure 2:**
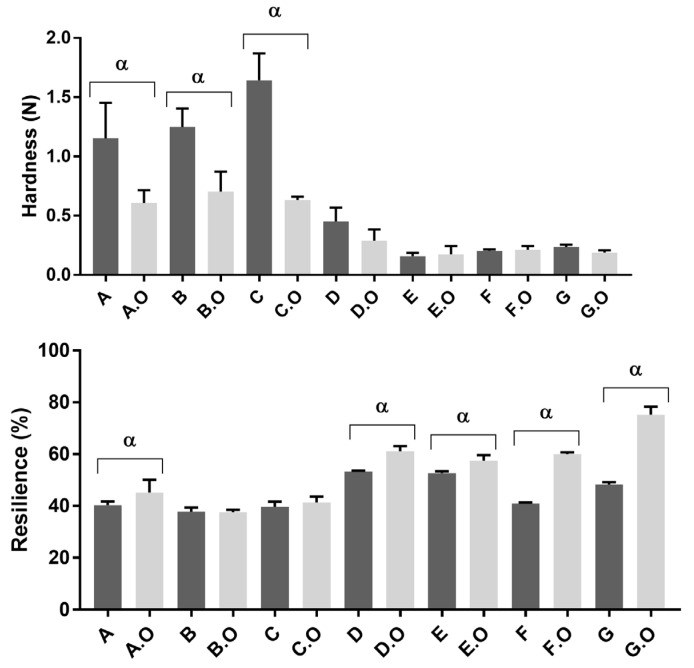
Study of textural properties of vaginal sheets: hardness (N)—part 1 and resilience (%)—part 2. α represents statistically significant difference between base formulation and formulation with oil (*p* < 0.05).

The concentration of polymer and plasticizer and/or their respective proportions influence the hardness and resilience of the formulations [20]. Vaginal sheet C was harder than B, so the inclusion of lactose produced a greater hardness than SSA (Figure 2).

The addition of oil to the hardest base formulations (A, B, C) resulted in a hardness decrease (statistically significant *p* < 0.05, Tukey’s multiple comparisons test). On the other hand, formulations E, F and G were less hard. These formulations have the lowest content of gelatine, despite containing other polymers such as HPMC (1% *w/w* gel for E and G) and PVA (2.4% and 1.2% *w/w* gel for E and F, respectively). The incorporation of TCEO in base formulations caused an increase in resilience (statistically significant for A, D, E, F and G, *p* < 0.05, Tukey’s multiple comparison test). Among the vaginal sheets with TCEO, G.O was the most resilient, followed by vaginal sheets D.O, E.O and F.O. This analysis of textural properties was in accordance with the results of folding endurance and the subjective evaluation of sensorial properties. As previously described, the resistance and the flexibility of the vaginal sheets influenced the ease of insertion and the perception of users [20]. Therefore, these results support preclinical characterization of vaginal dosage forms to predict their performance for in vivo applications. Users’ preferences should not be disregarded while developing vaginal dosage forms since these may dictate therapeutic adherence.

### 2.7. Bioadhesion

The rational design of the vaginal sheets included the selection of bioadhesive polymers that promote an intimate and prolonged contact of the sheets with epithelium [21,22,31,69,70,71], while absorbing and gradually dissolving in vaginal fluids. The dilution of the vaginal sheets in vaginal fluid may result in a gel which is expected to also present these bioadhesive properties, avoiding leakage and messiness.

**Figure 3:**
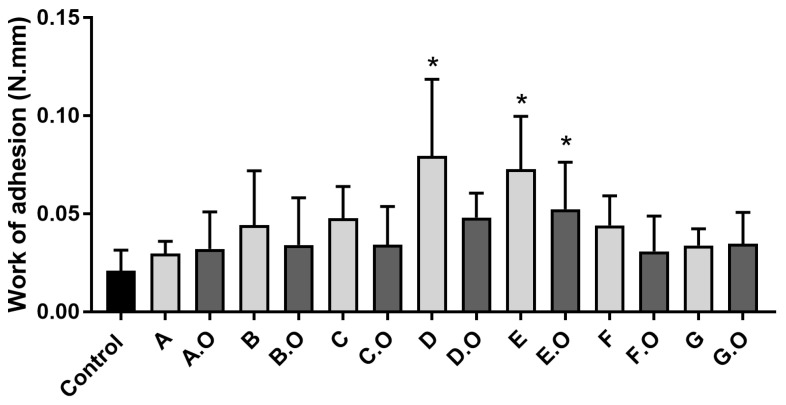
Evaluation of bioadhesive profile of vaginal sheets (work of adhesion (N.mm)). Individual columns and vertical bars represent mean and SD values, respectively (n = 6); * represents statistically significant difference from control (*p* < 0.05).

Among the base formulations, D was the most bioadhesive and among vaginal sheets with TCEO, formulation E.O was the most bioadhesive, followed by D.O (Figure 3).

These results are explained by the rational design of the formulations, since D and E include bioadhesive polymers: chitosan and HPMC. The addition of TCEO to the base formulations promoted changes in the interactions between the vaginal sheets and the vaginal epithelium, so the bioadhesive profile was altered (increased or decreased). Ideally, the vaginal sheet should be as bioadhesive as possible to be retained in the vaginal cavity long enough to absorb the excess vaginal fluids while releasing the active antimicrobial ingredient (TCEO).

### 2.8. Stability Studies

At room temperature, there was no significant change regarding vaginal sheet weight, indicating that no significant gain (absorption) of water was observed throughout the study (Supplementary Materials S3).

At 5 °C, the vaginal sheets were slightly increased in weight. This result may be due to moisture absorption from the cabinet, since the vaginal sheets were freeze-dried. At 40 °C, the vaginal sheets decreased in weight, which can be explained by the evaporation of residual water and eventually due to evaporation of the TCEO.

For vaginal sheets stored at room temperature and 5 °C, no changes in colour, odour, texture, hardness or malleability were detected, with the exception of vaginal sheet E.O which presented yellow spots after storage under both conditions. Vaginal sheet E.O is the only composition containing propyleneglycol together with glycerine as the plasticizer and additionally contains a mixture of gelatine, HPMC and PVA as polymers, so its composition is very different from the other vaginal sheets. It presented high and fast absorption capacity and completely disintegrated into a gel in that test. It can be hypothesized that for this formulation, some extent of absorption of TCEO might have occurred during the coating procedure which could impair a homogeneous distribution of this active substance on the surface. Eventually this heterogeneous coating was noticeable after storage.

Base formulations stored at 40 °C were slightly yellow, harder and less flexible, compared to t0 (after production). Vaginal sheets with EO were found to be more yellow (dark yellow) after storage indicating that some oxidation may have occurred and vaginal sheets C and C.O turned brown possibly due to the degradation of lactose contained in both of these sheets. To overcome these limitations, the addition of an antioxidant to these formulations might be considered in the future. Nevertheless, oxidation of essential oils has been shown to be highly dependent on temperature and to vary with the specific composition of the essential oil [72]. Therefore, although accelerated stability was performed at 40 °C in view of predicting longer term storage stability at room temperature (according to the Arrhenius equation), it is possible that the extent of oxidation observed at 40 °C does not directly correlate to oxidation occurring at room temperature.

Vaginal sheets with TCEO stored at 40 °C for 3 months still presented an odour typical of TCEO but was less intense compared to t0, possibly because some of the volatile components of TCEO may have volatilized [73]. Furthermore, in D and D.O sheets the characteristic chitosan odour was altered. Moreover, the vaginal sheets with TCEO were harder and less flexible, making it difficult to separate the vaginal sheet from the aluminium foil used for storage.

There was no significant variation in pH after dilution in mVFS for vaginal sheets stored at room temperature (Supplementary Materials S4). In this condition, all formulations presented pH variations from timepoint 0 below 0.2 pH units (that is a variation accepted for duplicate measurements). Similar observations were obtained for vaginal sheets stored at 5 °C (variation values were below 0.24). On the contrary, vaginal sheets stored at 40 °C exhibited an overall increase in pH (more than 0.2 pH units) for all formulations except for base formulations A and D. Formulation D.O exhibited an increase of 0.27 in pH from t0 (much lower than those observed for other vaginal sheets that ranged from 0.34 to 0.57). These results show that formulation D.O was the most stable regarding pH at accelerated conditions that may predict a more stable behaviour for prolonged periods at room temperature. Nevertheless, this parameter should be closely monitored throughout long-term (confirmation) stability studies at room temperature.

In short, all vaginal sheets were stable at room temperature for three months, but changes occurred at other storage temperatures. The obtained results highlight the importance of choosing a sealed package for the vaginal sheets (since they are produced though a freeze-drying process and they have been coated with TCEO) and the temperature conditions, with higher temperatures having a large influence. The data from the accelerated conditions point to higher stability of formulation D and D.O compared to the other formulations, despite the occurrence of oxidation reactions. These might be due to the essential oil and it is probable that they are related to higher temperatures (40 °C) making it difficult to extrapolate for long-term stability at room temperature.

### 2.9. Quantification of TCEO Components When Incorporated in Vaginal Sheets

Since vaginal sheet D.O presented the most promising technological characteristics (higher buffer capacity, higher absorption capacity, adequate textural properties) and one of the bests bioadhesive profiles, it was the prototype selected for the following characterization: quantification of active, safety and efficacy studies.

Incorporation rate of the TCEO into vaginal sheet D.O was 96.4% ± 2.9 considering the vaginal sheet as a unitary dosage form (i.e., considering the vaginal sheet as a whole) (Table 4).

Carvacrol concentration significantly decayed in the vaginal sheet D prepared by incorporation of the artificial mixture of carvacrol + linalool, independently of the storage conditions. Vaginal sheets prepared by incorporation of TCEO were apparently more stable during storage (regarding carvacrol concentration decay) than those prepared by incorporation of the artificial mixture of carvacrol + linalool through coating. These results may indicate that other components (minor components) of TCEO can contribute to higher stability, preventing volatilization.

In vaginal sheet D.O prepared by incorporation of TCEO through coating (% *w/w*), the decrease in carvacrol concentration after storage at 5 °C for 3 months was negligible (−1.91%). On the other hand, storage at 40 °C significantly decreased carvacrol concentration, proving that this high temperature caused the degradation or volatilization of volatile compounds. This result is further supported by the gravimetry results that showed loss of weight for this vaginal sheet (Supplementary Material S3). Therefore, we propose that for this prototype a sealed primary package should be selected and the products should be stored at room temperature or 5 °C, since the decrease of carvacrol after 3 months of storage at these temperatures was low. These results indicate that this vaginal sheet with TCEO is stable over time and that the three-dimensional network conferred by the freeze-drying of the gel effectively helped retain the volatile compounds of the EO, preventing their volatilization. Longer stability studies should be conducted for extrapolation of expiration dates of the final product.

### 2.10. Cellular Toxicity

**Figure 4:**
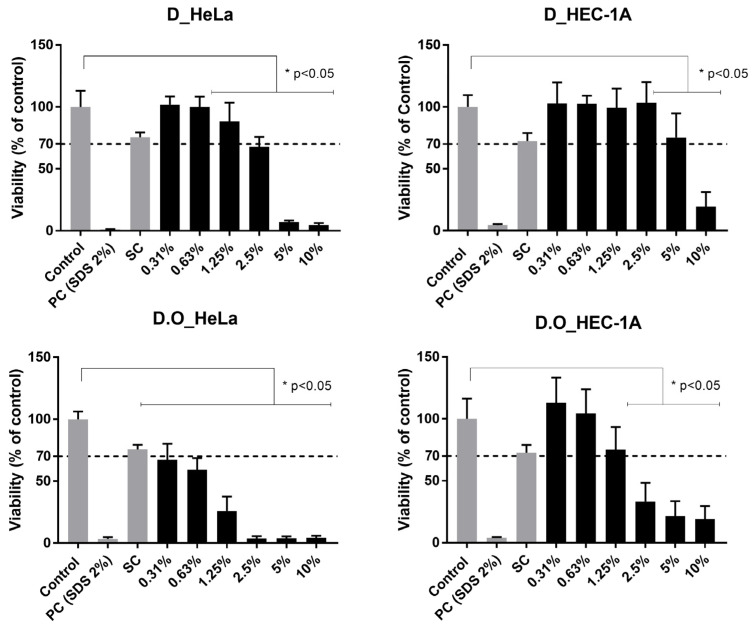
Cellular viability profile (MTT assay) of base formulation D tested in HeLa cell line and HEC-1A cell line and formulation D with oil tested in HeLa cell line and HEC-1A cell line. Cell viability is defined as percentage of the control treated only with culture media. Results are presented as the mean and standard deviations from 3 independent experiments. * represents statistically significant difference from negative control (*p* < 0.05).

As expected, treatment with the positive control resulted in a cell viability lower than 5% and the solvent controls results showed no significant reduction of cell viability. A dose-dependent toxicity of the vaginal sheets was observed (Figure 4). The base formulation D was non-toxic for the HeLa cell line at concentrations of 0.31%, 0.63% and 1.25% *w/v* and non-toxic at 0.31% to 5% for the HEC-1A cell line but revealed significant toxicity at higher concentrations for both cell lines. The vaginal sheet with TCEO (1% *w/w*) was non-toxic for HEC-1A at concentrations of 0.31%, 0.65% and 1.25% (corresponding to 0.031%, 0.0063 and 0.0125% of TCEO).

Using the same cell lines, the cytotoxicity of vaginal sheet D.O can be compared to the cytotoxicity of Dalacin V^®^ (a commercial semi-solid product also used for the treatment of BV) reported by Machado et al. [74]. Dalacin V^®^ was toxic, presenting lower viability than vaginal sheet D.O in both cell lines, including at lower concentrations of the formulation. Moreover, Dalacin V^®^ was also toxic when tested in an ex vivo model, exhibiting viabilities of about 20% at product concentrations of 5% and 20% *w/v* [74]. Thus, it is possible that these models are too sensitive for the effects of such products. Still, these are very important for comparative purposes.

The cell lines included in this cytotoxicity study represent two different epithelia of the female genital reproductive tract. Comparing cell lines, we can observe that the assays performed using HEC-1A cell lines presented higher viabilities than those performed using HeLa cells. HeLa cells were shown to be more sensitive to contact with the formulation, compared to HEC-1A. These results are in concordance with previous studies [74]. Gali et al. studied the toxicity of some excipients and active pharmaceutical excipients using various cell lines and only slight differences in sensitivity among the tested cell lines were observed. They reported that HEC-1A formed a stratified epithelia with a thickness of 4 to 5 cell layers (representative of intermediate between the endo- and ectocervical epithelia) and it could lead to lower toxicity compared to monolayer epithelia [56]. Therefore, it is vital to include different cell lines in the toxicity study of pharmaceutical products to achieve a more complete safety profile in the preclinical stage.

### 2.11. Vaginal Irritation—SkinEthic^TM^ Reconstructed Human Vaginal Epithelium Model

**Figure 5:**
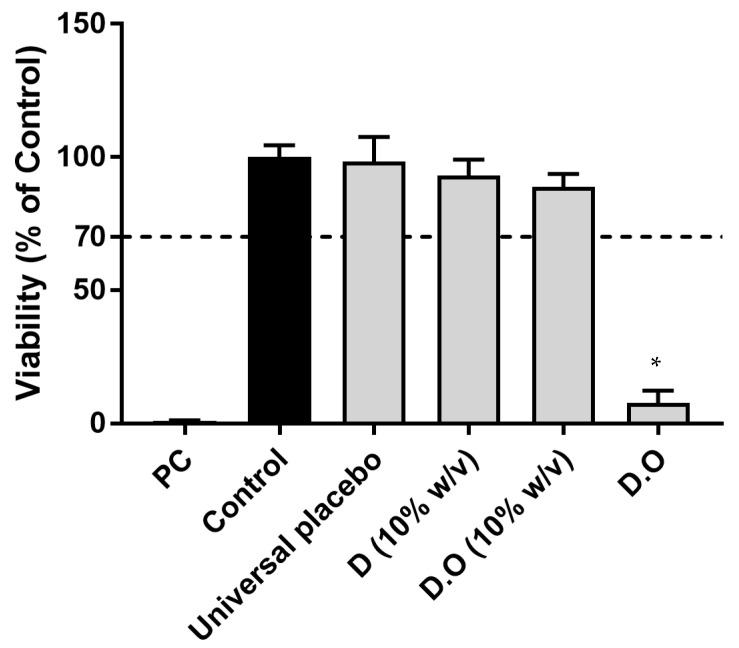
Cellular viability evaluated on Reconstructed Human Vaginal Epithelium model (MTT assay) of vaginal sheet D (10% *w/v*), vaginal sheet D with TCEO (10% *w/v* dissolution), vaginal sheet D with TCEO directly applied and universal placebo. Results are presented as the mean and standard deviation from 3 independent experiments. * represents statistically significant difference from negative control (*p* < 0.05).

Due to the higher sensitivity of the cellular model, further cytotoxicity testing was performed in a three-dimensional model with histological resemblance to the vaginal epithelium (Figure 5).

In both models (cellular and tissue), D (dissolved at 10% *w/v*) presented a higher viability than D.O. (10% *w/v*) indicating that TCEO contributes to the overall toxicity of the product at this concentration.

Vaginal sheet D (10% *w/v*) and D.O (10% *w/v*, corresponding to 0.1% *w/v* of TCEO) were biocompatible according to this model. Compared to the universal placebo, vaginal sheet D.O was significantly more toxic. This direct application over the tissue corresponds to an extreme testing procedure assumed as a worst case scenario. However, *in vivo*, vaginal sheet is not designed to be in contact with the vaginal epithelium during 24 h, since it gradually absorbs the vaginal simulant (that was not applied proportionally in this test), and becoming diluted by it. Therefore, in vivo, this toxicity may not occur, since the vaginal sheet will not be in contact with epithelium undiluted over this extended period. Intermediate toxicity may be expected when considering that only part of the formulation may swell through this contact. It should be considered that this formulation is not designed for chronic use and that the overall environment of the vaginal cavity is altered in BV.

### 2.12. Vaginal Irritation—Hen’s Egg Test-Chorioallantoic Membrane Assay (HET-CAM)

Vaginal sheets D and D.O were also tested in fine particles and diluted in saline solution (NaCl 0.9%). Vaginal sheets D and D.O tested in fine particles were defined as severe irritants. The hard nature and irregular and pointed shape of the particles may have influenced these results. Thus, the irritation potential obtained may result not only from the irritation caused by the constituents of the formulation (such as the hygroscopic nature of glycerine and gelatine), but also from a mechanical effect of the damage to the vessels when the sample is removed for viewing the endpoints, since there was adherence of the fine particles to the CAM [22]. Moreover, when the vaginal sheets were divided into fine particles, the superficial area was obviously significantly higher than the entire sheet and then the hygroscopic and hypertonic characteristics of formulations (conferred mainly by the gelatine and glycerine) become more evident, and it can lead to higher irritant potential. To confirm this influence on the results, sheets D and D.O were diluted in saline solution, which was a negative control since it is considered to be a non-severe irritant [75,76]. Therefore, the irritant potential obtained in these samples will result only from the effect of the sheets’ components at the tested final concentration. Vaginal sheets D and D.O diluted in saline solution (at 10% *w/v*) were considered as non-severe irritants (IS 5 ± 0 for D and 8 ± 0 for D.O) (Table 5). Concordant results were found with vaginal sheet D and D.O (10% *w/v*) using the tissue model. A concentration-dependent toxicity may be related to the results obtained.

For vaginal sheet D diluted in a saline solution, lysis (slight and limited) was observed as an outcome at the 0.5 min timepoint. For vaginal sheet D with TCEO, lysis was observed at the 0.5 min timepoint and haemorrhage also occurred at the 5 min timepoint. Again, these results show that the addition of TCEO to the base formulation increased the irritant potential.

### 2.13. Evaluation of the Vaginal Sheet D.O Efficacy against Gardnerella Species Biofilms

We tested the efficacy of vaginal sheet D.O against Gardnerella spp. biofilms, to simulate a scenario closer to in vivo conditions, since the polymicrobial dense and structured biofilms associated with BV are known to protect bacterial cells from the activity of antimicrobials, contributing to therapeutic failure. BV polymicrobial biofilms are primarily constituted by Gardnerella clusters that initially adhere to the vaginal epithelium and then facilitate the association and growth of other bacteria including *F. vaginae*, *Mobiluncus* spp. and *Prevotella* spp. with a synergic effect in the reestablishment of infection [7,12,77,78]. Since, to date, the role of each species of Gardnerella in the reestablishment of infection is not totally clarified (mostly due to the recent classification of species previously referred to as *Gardnerella vaginalis*), we included four species of Gardnerella for a more complete study considering several possible preponderant BV-causing bacteria [13]. Therefore, we evaluated the efficacy of the vaginal sheet containing TCEO on *Gardnerella* spp. cells organized in biofilms. The tested concentrations of the TCEO component were 0.32 µL/mL and 0.08 µL/mL, and were selected considering previous studies and the cytotoxicity results on 3D models obtained in this study.

**Figure 6:**
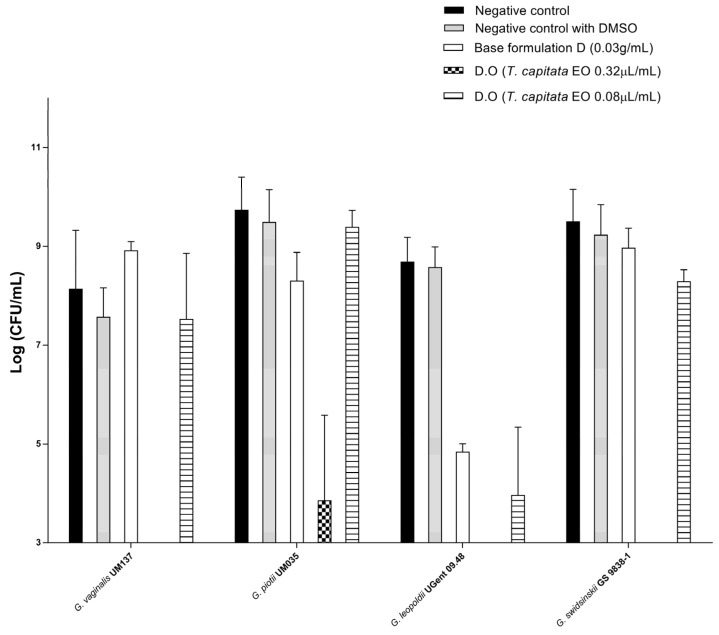
Effect of vaginal sheet D.O with TCEO on biofilms of four different species of *Gardnerella* at 0.32 µL/mL, 0.16 µL/mL and 0.08 µL/mL.

Our results showed that vaginal sheet D.O at 0.32 µL/mL of TCEO was able to significantly reduce the bacterial load of all tested *Gardnerella* species, being less efficient with *G. piotti* (Figure 6). For all strains except *G. piotti*, vaginal sheet D.O at 0.32 µL/mL of TCEO was able to reduce the number of CFUs below the limit of detection/quantification of the method. Our results from the vaginal irritation study using the SkinEthic^TM^ Reconstructed Human Vaginal Epithelium model indicated that this concentration (0.32 µL/mL of TCEO) was biocompatible. For *G. leopoldi*, 0.08 µL/mL was still able to reduce bacterial load. The goal of this study was not specifically to define the MLC of the formulation but to assess its ability to reduce the viability of the cells below the limit of quantification with the defined dose of TCEO that was not toxic to the 3D model. For most of the tested strains a dose–response curve was clearly observed. The *Gardnerella* species tested showed different sensitivities to vaginal sheet D.O. This fact should be considered in the rational design of new products being particularly relevant in the context of BV aetiology, since the BV-related biofilms are polymicrobial. Thus, the concentration of TCEO to be incorporated into the drug delivery system should be effective against several BV-related bacteria to guarantee the success of the treatment. 

Surprisingly, the base formulation was not active towards the biofilms, showing that chitosan nor the buffer solution significantly contributed to the overall efficacy but, indeed, some species may be sensitive to these effects. This is especially important since the polymicrobial biofilm of BV is the envisaged target. Additionally, it is important to highlight that the composition defined for the base intends to target the disease and not only the pathogen. For example, decreasing the pH of the vagina may be important to promote the reestablishment of the vaginal flora through beneficial growth of the protective microbiota. Additionally, absorption of fluids may enhance the effect of the EO delivered through the formulation. The concentration of the formulation that corresponds to 0.32 µL/mL of TCEO is 0.03 g/mL. This is much lower than the concentration assessed for compatibility with the 3D model (0.1 g/mL of base formulation corresponding to 1.07 µL/mL). Furthermore, the dose showed to be biocompatible corresponds to a 10% dispersion of the final product. These results may indicate that the concentration of the TCEO in the vaginal sheet is too high. By decreasing the concentration of TCEO, the effect of the active substance should be maintained, while decreasing inherent toxicity of the base.

## 3. Conclusions

We developed a TCEO vaginal drug delivery system through rational design to potentiate the treatment of BV through complementary and synergic actions of therapeutic agent and excipients. The vaginal sheets with TCEO were developed to promote immediate relief of the most significant symptom of this infection and to re-establish a healthy vaginal environment, while simultaneously allowing the potent natural active ingredient TCEO to act directly on the BV-related bacteria.

Vaginal sheet D.O composed of water, lactic acid, sodium lactate, gelatine, glycerine and chitosan coated with TCEO presented promising technological characteristics and the best predictable in vivo performance. D.O presented the highest buffer capacity and ability to absorb mVFS among all vaginal sheets with oil and showed one of the best bioadhesive profiles. Moreover, vaginal sheet D.O exhibited flexibility that allow it to be easily rolled and handled without breaking and a structure (hardness and thickness) that allows easy insertion.

This formulation was also able to hinder biofilm cells culturability of BV pathogens.. These results show that this product acts simultaneously and immediately in relieving the preponderant symptom (abundant vaginal discharge with unpleasant odour) and in inhibiting the pathogens. Effective concentrations were much lower than the concentration tested as biocompatible in the toxicity studies (0.03 g/mL for efficacy and 0.1 g/mL tested as biocompatible). Dose-dependent toxicity was observed for this vaginal sheet that indicates that the concentration of TCEO may have to be reduced for the sake of safety. Since this product was developed for short-term treatment, the obtained preclinical results show that vaginal sheet D.O is promising as an alternative treatment for BV.

## 4. Materials and Methods

### 4.1. Materials

For the preparation of vaginal sheets the following excipients were used: glycerine (Acofarma, Madrid, Spain), gelatine (Acofarma, Madrid, Spain); lactic acid 90% (VWR, Rosny-sous-Bois, France), sodium lactate 50% (VWR, Rosny-sous-Bois, France), sodium sulphate anhydrous (Labchem, Zelienople, PA, USA), lactose (Fagron, Terrassa, Spain), low molecular weight (50–190 kDa) chitosan (Sigma-Aldrich, Schnelldorf, Germany), hydroxypropyl methylcellulose (Methocel K100, viscosity 100,000 cP, 2% aqueous solution) (Dow, Midland, MI, USA), propylene glycol (Labchem, Zelienople, PA, USA), PVA 115000 (VWR, Rosny-sous-Bois, France) and MilliQ water (Sigma-Aldrich, Schnelldorf, Germany) (obtained in-housethrough a Merck Milli-Q^®^ Reference equipment).

The aerial parts of *Thymbra capitata* plant were collected at the flowering stage in Lagoa, Algarve (south of Portugal). The oils were isolated by hydrodistillation for 3 h, using a Clevenger-type apparatus according to the European Pharmacopoeia [79]. The oils were preserved in a sealed vial at 4 °C.

The vaginal simulant was prepared with following reagents: sodium chloride (JT Baker, Phillipsburg, NJ, USA), potassium hydroxide (VWR, Rosny-sous-Bois, France), calcium hydroxide (Acros Organics, Morris Plains, NJ, USA), bovine serum albumin (Sigma, Schnelldorf, Germany), lactic acid (Sigma, Schnelldorf, Germany), acetic acid (Fischer Scientific, Waltham, MA, USA), glycerol (Acofarma, Madrid, Spain), urea (VWR, Rosny-sous-Bois, France), glucose (VWR, Rosny-sous-Bois, France) and porcine gastric mucin type II (Sigma, Schnelldorf, Germany).

For the preparation of universal placebo, hydroxyethylcellulose (Ashland, OR, USA), sodium chloride (Honeywell fluka, Morris Plains, NJ, USA) and sorbic acid 99% (Alfa Aesar, Haverhill, MA, USA) were used.

Other reagents included: Dulbecco’s Modified Eagle Medium F12 (DMEM F12) (Gibco, New York, NY, USA), Roswell Park Memorial Institute (RPMI) 1640 medium (Biowest, Buffalo, NY, USA), sodium bicarbonate (Sigma, Schnelldorf, Germany), 3-(4,5-dimethylthiazol-2-yl)-2,5-diphenyltetrazolium bromide (MTT) (Alfa Aesar, Kander, Germany), foetal bovine serum (FBS) (Sigma, Schnelldorf, Germany), penicillin and streptomycin (Sigma, Schnelldorf, Germany), phosphate-buffered solution (PBS) (VWR, Rosny-sous-Bois, France), sodium dodecyl sulphate (SDS) (VWR, Rosny-sous-Bois, France), dimethyl sulfoxide (DMSO) (Fisher Chemical, Loughborough, UK), trypsin/EDTA solution (Fischer Scientific, Waltham, MA, USA), 2-propanol (Honeywell, Charlotte, NC, USA), sodium chloride (JT Baker, Phillipsburg, NJ, USA), sodium hydroxide (VWR, Rosny-sous-Bois, France), carvacrol (Sigma-Aldrich, Louis, MO, USA), ρ-cymene (Sigma-Aldrich, Louis, MO, USA) and linalool (Sigma-Aldrich, Louis, MO, USA).

### 4.2. Rational Design of Vaginal Sheets with TCEO

Vaginal sheets were developed based on our previously reported protocol with further modifications in polymer composition [20]. The present study represents the application of this dosage form to the treatment of BV by improving its ability to contribute to the overall efficacy and by incorporating TCEO as an active substance. The optimized method of production entails freezing vaginal sheets at −80 °C overnight before freeze-drying for 24 h using a Scanvac CoolSafe™ freeze drier (temperature reached −110 °C; pressure 0.019 hPa) to develop vaginal sheets that were then coated with *Thymbra capitata* EO [20].

Gelatine was used as the main macromolecule in all formulations since it is biocompatible, non-toxic and widely used in a variety of pharmaceutical formulations. It presents hygroscopic properties which allow it to absorb the abundant vaginal fluid [20,22,31]. Gelatine has already been used as an excipient to develop vaginal films [80]. Dolci et al. prepared gelatine-based films for the vaginal delivery of econazole to treat vulvovaginal candidosis.

The formulations included bioadhesive polymers commonly used in vaginal films, such as PVA, HPMC and chitosan to promote the efficient contact with the epithelium to avoid leakage [21,22,23,24,25,26,27,28,29,30,31,32,33,34,35,36,37,38] Furthermore, chitosan can contribute to the disruption of BV-related biofilms, and improve the treatment with metronidazole when used as an excipient to formulate liposomes [14,39,40,41,42,43,44,45,46].

The pH buffer solution (acid lactic/sodium lactate) was used to promote restoration of vaginal pH upon administration [21,22,31,32]. Two different strengths of buffer were tested.

Powdered active substances were included in vaginal sheets: lactose (inert powder) and anhydrous sodium sulphate (ASS) which present hygroscopic properties.

Glycerine and propylene glycol were used as plasticizers. Both are commonly used in vaginal products, including films, and are considered safe, although at high concentrations they can confer high osmolality to formulations which leads to toxicity [22,50,51,52,53,54,55]. Propylene glycol showed an higher potential to cause toxicity [56]. Therefore, glycerine was preferentially used as the plasticizer.

### 4.3. Preparation of Vaginal Sheets with TCEO

To prepare vaginal sheets, homogeneous gels containing buffer solution, gelatine, polymers (HPMC, PA and chitosan) and plasticizers (glycerine and propylene glycol) were prepared according to Table 6. For the preparation of gels, lactic acid/sodium lactate buffer was first heated to 50 °C (in a water bath) and then the plasticizers were added. Gelatine was dissolved in the mixture, and all the remaining constituents were incorporated. To prepare formulation D, chitosan was first dissolved in lactic acid/sodium lactate buffer, that is, before the addition of plasticizer and gelatine. To prepare formulations E and F, PVA was first dissolved in buffer by heating in a water bath at 90 °C for 60 min. Mechanical stirring with a helical stirrer (Heidolph RZR 2041 (Nurembergue, Germany)) at low speeds of rotation was performed to obtain homogeneous gels. The gels were centrifugated at 800 rpm for 5 min to remove trapped air bubbles. After centrifugation, 5× *g* of each gel was poured into standard small plastic Petri dishes (5.5 cm diameter). Formulations were allowed to cool down and were then frozen at −80 °C overnight. The formulations were then freeze-dried for 24 h using a Scanvac CoolSafe™ freeze drier (Zwijndrecht, The Netherlands) (temperature reached −110 °C; pressure 0.019 hPa).

TCEO was added to one surface of the vaginal sheet as a coating to a final concentration of 1% *w/w* (weight of TCEO/weight of vaginal sheet). For this purpose, TCEO was spread with a spatula on the upper surface of the base formulations. TCEO was mainly composed of carvacrol (73.9–80%). This coating method was used at the laboratory scale due to handling of very low amounts of material. At the industrial scale, the oil could be sprayed using calibrated equipment with a constant flow that allows for an exact dosage of the active.

The dimensions of 7 × 2.4 cm was previously proposed for vaginal sheets, corresponding to the mean dimension of the human vagina [20]. Throughout this study, the formulations were prepared and tested using the shape of the mould selected for freeze drying (circular shape, 5.5 cm diameter), resulting in smaller portions of vaginal sheets (for laboratory-scale purposes).

### 4.4. Freeze Drying Efficiency

The conversion of gels into vaginal sheets was achieved through freeze dying. This process aims to eliminate water from the formulation. Freeze drying efficiency was calculated according to the follow expression, assuming that all weight loss was due to water removal:

(1) $$\mathrm{Freeze}\;\mathrm{drying}\;\mathrm{efficiency}\;(\%)=\frac{initial\;weight\;of\;gel-weight\;of\;sheet\;after\;freeze-drying}{weight\;of\;water\;in\;formulation}\times 100$$

### 4.5. Preparation of mVFS

The mVFS was prepared as described by Owen and Katz (with an addition of mucin) as follows: sodium chloride 3.51 g, potassium hydroxide 1.4 g, calcium hydroxide 0.22 g, bovine serum albumin 0.018 g, lactic acid 2.00 g, acetic acid 1.00 g, glycerol 0.16 g, urea 0.4 g, glucose 5.00 g and 15.00 g porcine gastric mucin type II were added to slightly less than 1 L of MilliQ water and stirred mechanically until complete dissolution [81]. The pH of the mixture was then adjusted to 5 using sodium hydroxide, to mimic the pH characteristic of BV (pathological condition), and the final volume was adjusted to 1 L.

Mucin was used to simulate the bioadhesion properties of vaginal fluid [71,82].

### 4.6. Sensorial Characteristics

The sensorial characteristics studied were colour, transparency (transparent, opaque, translucent), odour (odourless, characteristic odour, intensity of odour) and feel (soft, hard, flexible, very flexible). All evaluations of visual and sensory parameters were performed 24 h after the preparation of the formulation by the same operator. Observations were recorded and photographs were taken.

### 4.7. Thickness

The thickness was measured using a digital micrometre IP54 (Vogel, VWR) on three different locations (one centre and two side edges locations) of two independent vaginal sheets of each formulation.

### 4.8. Absorption Efficiency of Vaginal Fluid Simulant

The evaluation of the absorption efficiency of the vaginal fluid simulant was performed using portions of vaginal sheets with dimensions of 1.75 × 0.6 cm (25% reduction of the proposed final size, maintaining the same proportion). For contact with vaginal fluid simulant not only the pH of the solution was adapted to the characteristics of this infection (pH 5) but also the volume of solution was increased in relation to physiologic conditions [81]. It is generally considered that 0.75 mL is present in the vagina at any given time in normal (physiologic) conditions [81]. While not specifically quantified in the literature, the excessive amounts of vaginal fluids in BV was considered to correspond to an excess of 50% of normal fluid volume. Therefore, 1.125 mL of fluid with pH 5.0 was considered to characterize BV vaginal fluid. For contact purposes with the vaginal sheet, the volume of fluid was reduced to 25% to keep the proportion determined by the reduction of the size of vaginal sheet. Thus, 0.281 mL of mVFS (25% of the vaginal fluid presented on vagina at any given time in BV), corresponding to 0.2834 g (based on density) was added to the upper side of the sheets (corresponding to 25% of the standard size). At determined timepoints, the vaginal sheet portions were cleaned to remove the unabsorbed mVFS that remained on the surface and were weighed (n = 3). Independent samples were tested for each timepoint.

The absorption efficiency of the vaginal fluid simulant was calculated according to the following equation:

(2) $$\mathrm{Absorption}\;\mathrm{efficiency}\;(\%)=\frac{weight\;at\;timepoint\;X\left(tx\right)-initial\;weight\left(t0\right)}{weight\;\left(mVFS\;added\right)}\times 100$$

### 4.9. pH and Buffer Capacity

The vaginal sheet was dissolved in a 1:20 ratio (sheet weight/solvent volume) at 37 °C. The solvents used were 0.9% *w/v* NaCl (since it exhibits low buffering capacity) and mVFS pH 5 (pH of BV vaginal fluids) [20].

The initial pH of the obtained dilutions was measured. Then, 20 μL of 1 N NaOH aqueous solution (corresponding to 0.02 meq NaOH) were added until the pH was equal to or greater than 9. Control assays were performed only with dissolution media (0.9% *w/v* NaCl or mVFS pH 5). The Absolute Buffering Capacity (ABC) defined as the amount of NaOH necessary to rise the pH one unit, and Relevant Buffer Capacity (RBC) defined as the amount of NaOH necessary to achieve a pH higher than 5, were calculated as previously described [20,53].

### 4.10. Textural Analysis: Hardness and Resilience

Hardness and resilience were measured through a TA.XT Plus Texture Analyser (Stable Micro Systems, Godalming, UK), as described by Machado et al. [20].

Hardness was determined as the Fmax exerted by a 2 mm needle shape probe (P2N, Stable Micro Systems, Godalming, UK) on the formulation placed on a heavy duty platform (test speed 3 mm/s, penetration distance of 0.2 mm, trigger force 0.05 N). For resilience measurements, a P2 (2 mm) flat probe was used and a ring platform prevented the movements of the formulation (test speed 3 mm/s, distance 2 mm, trigger force 0.05 N).

For each formulation, measurements were performed at three different points using two independent vaginal sheets (n = 6).

### 4.11. Bioadhesion

The bioadhesion of the vaginal sheets to ex vivo porcine vaginal tissue was measured through a texturometer TAXT Plus (Stable Micro Systems, Godalming, UK), according to a previously described method [20].

Circular portions of vaginal sheets with a 10 mm diameter were used. On the upper side of each circular portion, 53 μL of mVFS were added and were left in contact for 20 min immediately before the test. This volume was defined proportionally, considering the area of the vaginal sheet circular portions and the increased volume of vaginal fluid in BV [20,81]. The vaginal epithelium samples were hydrated with 20 μL of mVFS immediately before the beginning of the determination. The whole system was kept at 37 ± 1 °C by means of an oven (Stable Micro Systems, Godalming, UK) (Supplementary Materials S5).

The method details were as follows: bioadhesion mode, pre-test speed was 0.5 mm/s, trigger force 0.02942 N, test speed and post-test speed were 0.1 mm/s, contact/hold time was 180 s and the force applied was 2.5 N.

Each formulation was tested on six portions of different vaginal tubes (n = 6).

Bioadhesive profiles of vaginal sheets were expressed as work of adhesion (N.mm) and were compared to the control, consisting of a cellulose acetate membrane attached with double-sided adhesive tape and treated with the same procedure.

### 4.12. Stability Studies

Vaginal sheets were wrapped in aluminium foil and stored at 20–25 °C, 5 ± 3 °C and 40 ± 2 °C for 3 months. The storage temperatures were selected based on the International Council for Harmonisation of Technical Requirements for Pharmaceuticals for Human Use (ICH Q1A (R2)). The humidity was not controlled. We studied the changes in sensorial characteristics, gravimetry (variation of weight), pH after the dilution in mVFS and textural properties. Observations were recorded and photographs were taken. The results were compared to the initial characterization (t0).

#### Quantification of TCEO Components When Incorporated in Vaginal Sheets

The quantification of TCEO was based on carvacrol content in vaginal sheet D.O and was performed immediately after incorporation of TCEO (t0) and after storage for 3 months, under different temperature conditions. Meanwhile, vaginal sheets were prepared by incorporating a mixture of pure compounds, carvacrol and linalool (80:20) at 1% weigh/weight of sheet. These sheets, stored in the same conditions, were used to support the validation of the quantification procedures of carvacrol and linalool in the vaginal sheets. The incorporation rate of TCEO into vaginal sheets was calculated immediately after the incorporation (t0) of TCEO 1% *w/w* considering two independent batches (n = 1 for batch 1 and n = 3 for batch 2). Carvacrol was quantified by gas chromatography equipped with a flame ionization detector (GC-FID) after a suitable liquid–liquid extraction.

Briefly, vaginal sheets (unitary samples of 2 g) were roughly divided and submitted to extraction with 3 × 20 mL of a mixture of n-pentane/diethyl oxide (93:7) for 2 h, under continuous shaking. The extractive solutions were decanted and combined. The residual vaginal sheets were then mixed with 20 mL deionized water and 20 mL n-pentane/diethyl oxide (93:7) and kept under continuous shaking for 2 h. After centrifugation (6000 rpm × 3 min), the organic liquid phase was recovered and added to the previous solution. The volume of the extractive solution was reduced to less than 18 mL by distillation under reduced pressure (640 mbar) at room temperature; 10 mg of camphor was added as an internal standard [1.0 mL of a camphor solution at 10.0 mg.mL^−1^ in n-pentane/diethyl oxide (93:7)] and the final volume was adjusted to 20 mL with the solvent mixture.

Each sample was analysed by gas chromatography in a Hewlett-Packard 6890 gas chromatograph equipped with an FID and a SPB-1 (polydimethylsiloxane 30 m × 0.20 mm i.d., film thickness 0.20 µm) column. The oven temperature was set to raise from 70 °C to 190 °C (at a rate of 12 °C·min^−1^); injector temperature was set to 250 °C; helium was used as the mobile phase with a flow adjusted to maintain a linear velocity of 30 cm·s^−1^; 1 µL of sample was injected in splitless mode; FID temperature was set at 250 °C.

The quantification of carvacrol was performed by the internal standard method referring to the GC peaks’ raw areas (carvacrol and camphor peaks) in the samples and in a standard solution containing carvacrol at 0.7 mg/mL and camphor at 0.5 mg·mL^−1^ (as internal standard).

The Limit of Quantification for carvacrol was estimated as 7000 μg/g of sample.

### 4.13. Cellular Toxicity

#### 4.13.1. Epithelial Cells

HeLa (derived from human uterine cervical adenocarcinoma) and HEC-1A (derived from human endometrial adenocarcinoma) cell lines were obtained from the American Type Culture Collection (ATCC-LGC Promochem, Teddington, UK). The HeLa cell line was cultured in DMEM-F12 medium supplemented with penicillin (100 U/mL), streptomycin (100 mg/mL), and 10% FBS and is hereafter referred to as DMEM complete medium (passages 39–49). The uterine HEC-1A cells were cultured in RPMI 1640 supplemented with 100 U/mL penicillin, 100 mg/mL streptomycin, and 10% FBS, hereafter referred to as RPMI complete medium (Passages 50–55).

#### 4.13.2. Samples Tested

Cytotoxicity testing was performed for base formulation D and formulation D with TCEO. The sheets were dissolved to 10% *w/v* in complete culture medium containing 0.5% (*v/v*) DMSO for 4 h at 37 °C, and the dispersions were then vortexed for 3 min. Serial dilutions were then performed to 5%, 2.5%, 1.25%, 0.63% and 0.31% (*w/v*) in complete culture medium containing 0.5% (*v/v*) DMSO. Pure TCEO was diluted to 0.25%, 0.125%, 0.063%, 0.031%, 0.015% and 0.007% (*v/v*) in complete culture medium containing 0.125% (*v/v*) DMSO and vortexed for 3 min. The resultant emulsion was homogeneous, stable and was visually inspected for phase separation (that did not occur throughout the study timeframe).

### 4.14. Cytotoxicity Test (MTT Assay)

The MTT reduction assay was performed as previously described and according to ISO/EN 10993-5 for the in vitro evaluation of medical devices [83]. Cells were seeded onto 96-well plates (100,000 cells/mL) with complete culture media and were left to adhere for 24 h at 37 °C, under a 5% CO_2_ atmosphere. After obtaining a half-confluent culture, 100 μL of tested samples were used to treat the cells for 24 h. After this period, the cells were washed with PBS and incubated for 3 h with 50 µL of a 1 mg/mL solution of MTT reagent prepared in incomplete culture medium. Extraction of the formed formazan crystals was accomplished with 100 μL of 2-propanol for 15 min, through mild agitation on an orbital shaker, protected from light. A microplate spectrophotometer (Biorad xMark, EUA) was then used to measure the absorbances at 570 nm. Cells without any treatment (only culture media) were used as a negative control representing 100% viability reference for the products’ toxicity calculation. A positive control (SDS 2%) and solvent controls (SC: 0.5% DMSO in culture media for the assays of D and D.O, and 0.125% DMSO in culture media for the assays of EO) were included.

### 4.15. Vaginal Irritation-SkinEthic^TM^ Reconstructed Human Vaginal Epithelium model

For safety characterization, vaginal irritation was further studied in a commercial 3D human vaginal epithelium model (SkinEthicTM HVE/Human Vaginal Epithelium, provided by Episkin France), using the MTT test.

The inserts with tissues were visually inspected for integrity and individually transferred to 6-well plates (VWR, Portugal) pre-filled with 1 mL of Maintenance Medium (them, Episkin). The tissues were then pre-incubated overnight at 37 °C, ≥90% humidity, 5% CO_2_ (Binder APT.lineTM C150E2, USA).

Afterwards, the tissues were transferred to new 24-well plates (one plate per condition) containing 300 µL of Maintenance Medium, and then 30 µL of the liquid/semi solid test substances or controls were gently dispersed over the entire tissue surface. We tested the vaginal irritation caused by vaginal sheet D diluted in PBS 10% *w/v*; vaginal sheet D.O diluted in PBS 10% *w/v*; vaginal sheet D.O (undiluted, tested in direct contact with tissue through circular portions with the same diameter of tissues added to 10 µL of PBS); and universal placebo, used as a comparator/reference. PBS without Ca^2+^ and Mg^2+^ (DPBS, VWR) and SDS 1% *w/v*, were used as negative and positive controls, respectively.

Treatment was performed for 24 h at 37 °C, ≥90% humidity, 5% CO_2_. Afterwards, tissue viability was assessed by the MTT assay [50]. Briefly, the tissues were washed with PBS and gently dried. Then, they were transferred to a 24-well plate containing 300 µL per well of a 0.5 mg/mL MTT (Alfa Aeser) solution (in PBS, VWR) and incubated for 3 h, at 37 °C, ≥90% humidity, 5% CO_2_, protected from light. After this period, the tissues were transferred to a single 24-well plate containing 750 µL of isopropyl alcohol and an additional 750 µL was added at the top of each tissue to allow for full extraction of formazan for ≥2 h, in a sealed plastic bag, under agitation in a plate stirrer. The absorbances were then measured at 570 nm, using a microplate spectrophotometer (Promega GloMax^®^ Explorer System, Madison, WI, USA). The results were normalized to the viability of PBS-treated tissues (considered as 100% viability). Wells with isopropyl alcohol were used for background deduction which was applied to all absorbance values.

### 4.16. Vaginal Irritation—Hen’s Egg Test-Chorioallantoic Membrane Assay (HET-CAM)

The HET-CAM assay was performed on fresh fertile White Leghorn chicken eggs that were incubated at 37.8 ± 0.3 °C in a relative humidity of 58 ± 2% and under automatic rotation for 8 days (Corti AF-50 and Copele 30652, Spain). On the eighth day non-embryonated and non-viable (dead) eggs were discarded and viable eggs were incubated for one further day under the same conditions (but without rotation), as described by Palmeira-de-Oliveira et al. [76].

The HET-CAM assay (according to the ICCVAM—Recommended Test Method (NIH Publication No. 10-7553—2010) was used to test the potential severe vaginal irritation of the samples, since it allows the identification of severe irritants as an in vitro alternative to the in vivo Draize rabbit eye test [75] and was shown to be useful for vaginal irritation testing [76].

On day 9, eggs were taken out of the incubator and the shell was carefully opened [76]. The internal membrane was exposed and then hydrated with NaCl 0.9% (*w/v*) for a maximum of 30 min. The solution was decanted, and the membrane was carefully peeled off, without damaging the blood vessels. Then, 0.3 mL of base formulation D and formulation D.O diluted in saline solution (10% *w/v*) and control solvents and 0.3 g of base formulation D and formulation D.O finely divided were applied to the CAM (n = 3 eggs per sample), ensuring that at least 50% of the CAM surface area was covered. Saline solution was selected to dissolve the vaginal sheets since it is considered a non-severe irritant. The resultant viscous gels were thermostated at 37 °C before being applied. NaCl 0.9% (*w/v*) was used as a negative control and NaOH 0.1 N and SDS 1% were used as positive controls.

Observation of three endpoints were used for classification of the samples: haemorrhage (vessel bleeding), lysis (vessel disintegration) and coagulation (intra- and extra-vascular protein denaturation) at predetermined time intervals (0.5, 2, and 5 min). These observations were used for the calculation of the irritation score (IS (A)) (Supplementary Material S6). The irritancy classification was defined as non-severe irritant (IS 0–9) or severe irritant ( >9 to 21) [76].

### 4.17. Evaluation of Vaginal Sheet D.O efficacy against Gardnerella Species Biofilms

#### 4.17.1. Bacterial Growth Conditions

*G. vaginalis* UM137, *G. piotii* UM035, *G. leopoldii* UGent 09.48 and *G. swidsinskii* GS 9838-1 were grown on Columbia Agar Base medium (Liofilchem, Roseto degli Abruzz, Italy) supplemented with 5% (*v/v*) defibrinated horse blood (Oxoid Ltd., Hampshire, UK) for 48 h [7]. For each experiment, the bacterial species were grown in Brain Heart Infusion (BHI, Liofilchem) supplemented (sBHI) with 2% (*w/v*) gelatine (Liofilchem), 0.1% (*w/v*) starch (Panreac, Barcelona, Spain) and 0.5% (*w/v*) yeast extract (Liofilchem) and incubated for 24 h at 37 °C and 10% CO_2_ (Panasonic MCO-18AC, Bracknell, UK).

#### 4.17.2. Activity of Dissolved Vaginal Sheets on Gardnerella Species Biofilm

For biofilm formation, 24 h bacterial inoculums of *Gardnerella vaginalis* UM137, *G. piotii* UM035, *G. leopoldii* UGent 09.48 and *G. swidsinskii* GS 9838-1 were adjusted to a concentration of 10^7^ CFU/mL, determined by flow cytometry, as previously described [10]. Then, 1 mL of each suspension was dispensed on 24 well-plates (Orange Scientific, Braine-l’Alleud, Belgium) and incubated for 24 h at 37 °C and 10% CO_2_.

The vaginal sheet D.O was dissolved (at 10% *w/v* concentration), using sBHI medium containing 0.5% (*v/v*) DMSO to ensure proper solubility of the formulations. The required concentrations of the dissolved vaginal sheets were prepared in sBHI medium. After 24 h, the medium from the biofilms was removed and 1 mL of the correspondent vaginal sheet suspension was added to the biofilm and the plates were incubated for a further 24 h at the same conditions. A negative control was performed where the medium was replaced by fresh sBHI. Another control was included where the effect of DMSO was verified by replacing the medium at 24 h with sBHI with 0.5% DMSO. The effect of the vaginal sheet D without EO was also assessed using the same method applied to the EO-containing vaginal sheet. Vaginal sheet D was tested at a concentration of 0.03 g/mL that corresponds to the concentration of the base formulation at the higher concentration of D.O tested (TCEO 0.32 µL/mL).

After 48 h of biofilm formation, the medium was removed and the biofilm was washed once with NaCl 0.9% (*w/v*). Then, 1 mL of sBHI was added to each well and the biofilm was detached. A total of 100 µL of each condition was diluted in NaCl 0.9% (*w/v*) and serial dilutions were performed. Lastly, 10 μL of each dilution were plated on CBA plates and incubated for 72 h at 37 °C and 10% CO_2_. Colony Forming Unit (CFU) counts were performed and results were expressed as log CFU/mL. The experiments were repeated at least three times with technical duplicates.

## Figures and Tables

**Table 1:** Sensorial characteristics of vaginal sheets.

Formulation	Colour	Transparency	Odour	Feel to Touch
A	Very light yellow	Transparent	Odourless	Hard, flexible, smooth surface
A.O	Light yellow	Transparent	TCEO odour	Flexible, smooth surface
B	Yellowish white	Translucent	Odourless	Flexible, smooth surface
B.O	Very light yellow	Translucent	TCEO odour	Hard, flexible, smooth surface
C	Very light yellow	Transparent	Odourless	Flexible, smooth surface
C.O	Light yellow	Transparent	TCEO odour	Hard, flexible, smooth surface
D	Light yellow	Transparent	Slight shellfish odour	Flexible, smooth surface
D.O	Light yellow	Transparent	TCEO odour; slight shellfish odour	Very flexible, smooth surface
E	White	Opaque	Odourless	Soft, flexible, smooth surface
E.O	White	Opaque	TCEO odour	Soft, very flexible, smooth surface
F	Very light yellow	Translucent	Odourless	Soft, flexible, smooth surface
F.O	Very light yellow	Translucent	TCEO odour	Soft, very flexible, smooth surface
G	Pearl white	Opaque	Odourless	Soft, flexible, smooth surface
G.O	Pearl white	Opaque	TCEO odour	Soft, very flexible, smooth surface

**Table 2:** Thickness (mm) and pH after diluting vaginal sheets in vaginal fluid simulant with mucin (mVFS) pH 5 (1:10 *w/w*).

	Thickness (mm) (n = 6)	pH Dilution with mVFS pH5 1:10 *w/w* pH ± S.D (n = 3)
A	0.935 ± 0.081	4.72 ± 0.01
A.O	0.925 ± 0.025	4.48 ± 0.02
B	1.111 ± 0.176	4.63 ± 0.03
B.O	1.086 ± 0.072	4.47 ± 0.03
C	0.815 ± 0.039	4.61 ± 0.01
C.O	0.821 ± 0.018	4.45 ± 0.01
D	0.813 ± 0.046	4.67 ± 0.01
D.O	0.820 ± 0.023	4.52 ± 0.01
E	0.921 ± 0.041	4.43 ± 0.01
E.O	0.906 ± 0.012	4.38 ± 0.01
F	1.027 ± 0.023	4.52 ± 0.01
F.O	1.005 ± 0.009	4.40 ± 0.01
G	0.920 ± 0.024	4.55 ± 0.01
G.O	0.924 ± 0.010	4.40 ± 0.02

**Table 3:** Absorption efficiency (%) of mVFS of vaginal sheets over 24 h. Results are presented as mean value ± standard deviation (SD), n = 3. Bold values represent the maximum absorption (swelling) timepoint from which preparations start to lose their structure.

	10 min	30 min	1 h	3 h	5 h	7 h	11 h	24 h
**A**	21 ± 3	35 ± 1	54 ± 0	55 ± 2	59 ± 3	**63 ± 0**	59 ± 8	57 ± 1
**A.O**	29 ± 2	42 ± 2	42 ± 2	**53 ± 6**	46 ± 3	47 ± 5	47 ± 2	33 ± 1
**B**	29 ± 4	44 ± 4	54 ± 0	62 ± 3	66 ± 0	67 ± 7	**84 ± 14**	55 ± 1
**B.O**	28 ± 4	39 ± 3	45 ± 2	53 ± 2	**60 ± 2**	54 ± 4	45 ± 1	32 ± 0
**C**	22 ± 3	42 ± 7	39 ± 1	56 ± 3	53 ± 0	55 ± 7	**59 ± 14**	50 ± 1
**C.O**	29 ± 0	41 ± 2	44 ± 0	50 ± 6	50 ± 2	**53 ± 4**	52 ± 4	34 ± 1
**D**	25 ± 5	45 ± 5	47 ± 2	59 ± 12	63 ± 5	69 ± 2	**70 ± 1**	61 ± 0
**D.O**	40 ± 4	42 ± 8	49 ± 5	48 ± 1	56 ± 3	**63 ± 0**	45 ± 6	32 ± 0
**E**	**42 ± 9**	39 ± 5	32 ± 3	29 ± 10	10 ± 0	-	-	-
**E.O**	46 ± 7	**47 ± 2**	42 ± 5	35 ± 8	6 ± 2	-	-	-
**F**	20 ± 15	37 ± 7	40 ± 11	49 ± 4	**58 ± 0**	47 ± 1	47 ± 6	23 ± 0
**F.O**	36 ± 4	41 ± 3	53 ± 1	**56 ± 2**	55 ± 2	55 ± 3	55 ± 3	37 ± 5
**G**	31 ± 1	40 ± 11	44 ± 6	**47 ± 2**	47 ± 4	46 ± 10	47 ± 5	26 ± 11
**G.O**	38 ± 3	48 ± 2	48 ± 8	**52 ± 5**	51 ± 8	51 ± 4	47 ± 3	38 ± 3

The bold represents the timepoint, when each formulation began to dissolve in mVFS and gradually formed a gel.

**Table 4:** Evaluation of TCEO content (represented by carvacrol component) after 3 months of storage at 40 °C, 5 °C and room temperature (20–25 °C) for vaginal sheet D (preferred composition).

	Storage Conditions during 3 Months	Decrease of Concentration of Carvacrol after Storage from t0 (%)
**Vaginal sheet D** **Mixture Carvacrol + Linalool (0.8 + 0.2% *w/w*)**	5 °C	−41.44%
**Vaginal sheet D** **Mixture Carvacrol + Linalool (0.8 + 0.2% *w/w*)**	Room temperature (20–25 °C)	−82.72%
**Vaginal sheet D** **Mixture Carvacrol + Linalool (0.8 + 0.2% *w/w*)**	40 °C	−93.45%
**D.O** **TCEO (1% *w/w*)**	5 °C	−1.91%
**D.O** **TCEO (1% *w/w*)**	Room temperature (20–25 °C)	−16.83%
**D.O** **TCEO (1% *w/w*)**	40 °C	−56.26%

**Table 5:** Irritation Score and classification of samples. Results are presented as mean value ± standard deviation (SD), n = 3.

		IS (Mean ± Standard Deviation)	Classification
**Controls**	NaCl 0.9% (*w/v*)	0 ± 0	Non-severe irritant
	NaOH (0.1 N)	20 ± 1	Severe irritant
	SDS 1% (*w/v*)	10 ± 0	Severe irritant
**Tested product**	D (fine particles)	11 ± 1	Severe irritant
	D.O (fine particles)	11 ± 1	Severe irritant
	D 10% *w/v* in saline solution	5 ± 0	Non-severe irritant
	D.O 10% *w/v* in saline solution	8 ± 0	Non-severe irritant

**Table 6:** Qualitative and quantitative composition (% *w/w*) of gels used for vaginal sheets preparation.

	Water	LA (90% *v/v*)	Sodium Lactate (50% *v/v*)	Gelatine	HPMC	PVA	Glycerine	Propylene Glycol	ASS	Lactose	Chitosan
**A**	70.42	0.81	2.52	15.0			11.25				
**B**	67.54	0.79	2.42	15.0			11.25		3.0		
**C**	67.54	0.79	2.42	15.0			11.25			3.0	
**D**	63.65	1.56	4.79	13.5			15.0				1.5
**E**	81.82	0.95	2.93	4.0	1.0	2.4	6.0	0.9			
**F**	70.45	0.82	2.53	10.0		1.2	15.0				
**G**	70.65	0.82	2.53	10.0	1.0		15.0				

LA—lactic acid/sodium lactate buffer solution; HPMC—hydroxypropylmethylcellulose; PVA—polyvinyl alcohol; ASS—anhydrous sodium sulphate.

## Data Availability

Not available.

## Supplementary Materials

The following supporting information can be downloaded at: https://www.mdpi.com/article/10.3390/gels9040293/s1, Supplementary Materials S1: Freeze-drying efficiency, Supplementary Materials S2: General aspect of vaginal sheets, coating and handling. Legend: 1—vaginal sheet A; 2—vaginal sheet B; 3—vaginal sheet C; 4—vaginal sheet D; 5—vaginal sheet E; 6—vaginal sheet F; 7—vaginal sheet G; 8—Method of application of TCEO onto the surface of vaginal sheets after the freeze-drying process using a spatula; Supplementary Materials S3: Gravimetry (weight variation %) after storage for 3 months compared to t0, Supplementary Materials S4: pH after diluting vaginal sheets in mVFS pH 5 (1:10 *w/w*) after storage for 3 months. Results are presented as mean value ± standard deviation (SD), n = 3, Supplementary Materials S5: Illustration of the method for evaluation of bioadhesive profile of vaginal sheets on a texturometer using ex vivo porcine vaginal tissue. 1—Preparation of vaginal porcine epithelium. 2—A double sided adhesive tape allowed for circular portions of vaginal sheets attachment. 3—The whole system was maintained at 37 °C by means of an oven. 4—Porcine vaginal tissue was fixed using a mucoadhesion rig (A-MUC), avoiding its movement when the probe moves and allowing intimate contact between the formulation and the epithelium. Supplementary Materials S6: Irritation score calculation according to the endpoint at each time point.

## Abbreviations

ABC	Absolute Buffer Capacity
ASS	anhydrous sodium sulphate
BV	bacterial vaginosis
HPMC	hydroxypropylmethylcellulose
LA	lactic acid
MIC	minimum inhibitory concentration
MLC	minimum lethal concentration
mVFS	vaginal fluid simulant with mucin
PVA	polyvinyl alcohol
RBC	Relevant Buffer Capacity
spp.	several species
TCEO	*Thymbra capitata* essential oil
VFS	vaginal fluid simulant
