# Supplementary material for: Vaginal Sheets with Thymbra capitata Essential Oil for the Treatment of Bacterial Vaginosis: Design, Characterization and In Vitro Evaluation of Efficacy and Safety
Source: Gels. 2023 Apr 2;9(4):293. doi: 10.3390/gels9040293 (PMC10137747; doi:10.3390/gels9040293)
Supplement: Supplementary file 1 [file gels-09-00293-s001.zip › gels-2080591-supplementary.pdf]

Supplementary Materials

Vaginal Sheets with *Thymbra capitata* Essential Oil for the Treatment of Bacterial Vaginosis: Design, Characterization and *In Vitro* Evaluation of Efficacy and Safety

Mariana Tomás [1], Lúcia G. V. Sousa [2,3], Ana Sofia Oliveira [1], Carolina P. Gomes [1], Ana Palmeira-de-Oliveira [1,4], Carlos Cavaleiro [5,6], Lígia Salgueiro [4,6], Nuno Cerca [2,3], José Martinez-de-Oliveira [1] and Rita Palmeira-de-Oliveira [1,3,*]

S1. Freeze drying efficiency

Base formulation	Freeze drying efficiency (%)
A	98.5 ± 0.8
B	94.7 ± 1.9
C	97.9 ± 0.6
D	93.8 ± 3.0
E	96.4 ± 0.4
F	94.4 ± 1.2
G	96.5 ± 3.0

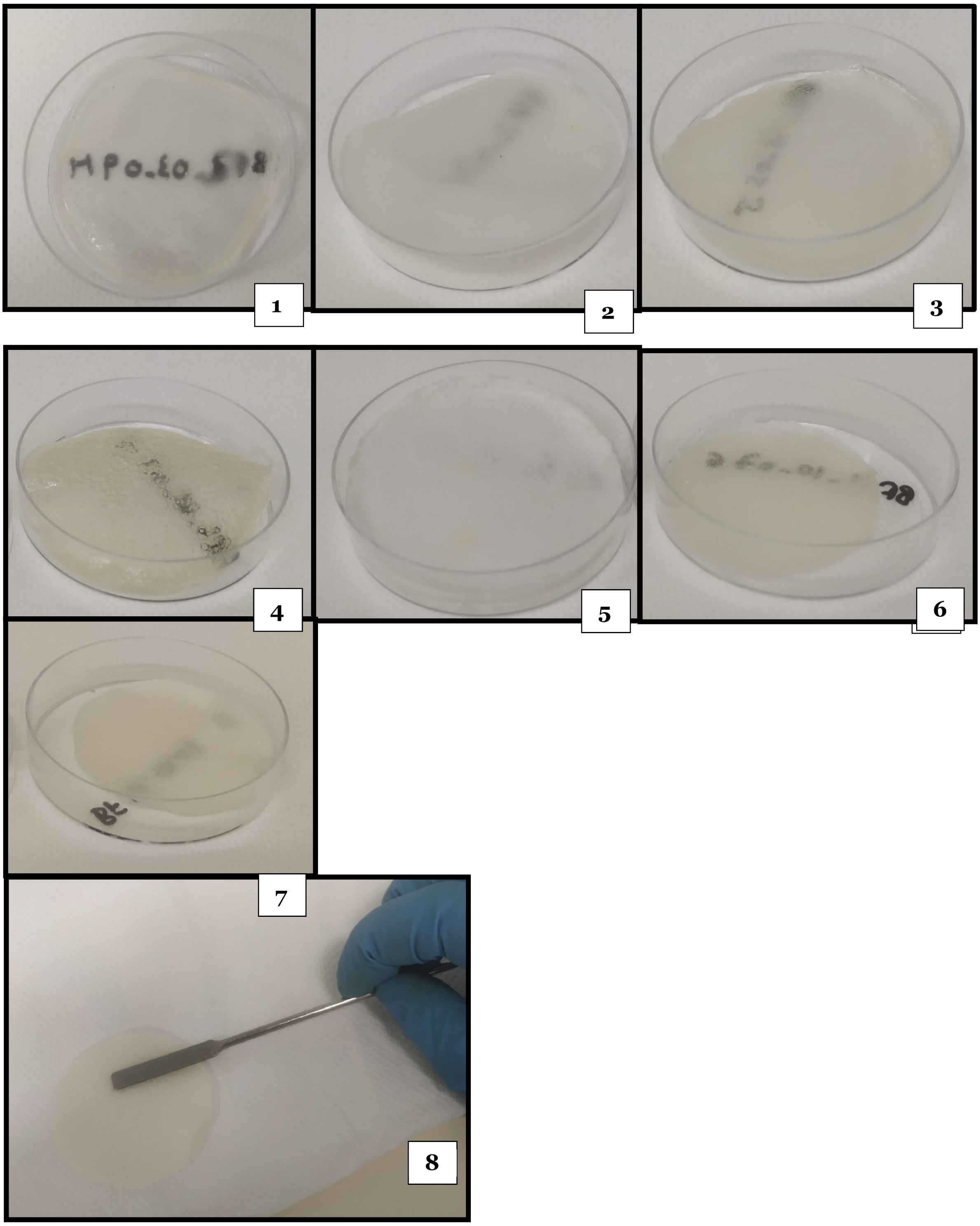

S2. General aspect of vaginal sheets, coating and handling. Legend: 1 – vaginal sheet A; 2 – vaginal sheet B; 3 – vaginal sheet C; 4 – vaginal sheet D; 5 – vaginal sheet E; 6 – vaginal sheet F; 7 – vaginal sheet G; 8 – Method of application/coating of TCEO on the surface of vaginal sheets after the freeze-drying process using a spatula;

S3. Gravimetry (weight variation %) after storage for 3 months compared to t0

Formulation	Variation of weight (%)		
	**TA**	**5 ºC**	**40 ºC**
**A**	-1.60	6.16	-1.12
**A.O**	-1.90	3.90	-5.88
**B**	1.35	5.88	-4.92
**B.O**	-1.18	4.19	-3.26
**C**	1.37	6.08	-3.51
**C.O**	-0.51	3.24	-7.97
**D**	1.29	8.12	-4.88
**D.O**	0.30	4.28	-7.81
**E**	2.98	7.83	-1.35
**E.O**	-0.86	5.93	-5.09
**F**	2.55	8.15	-2.07
**F.O**	0.60	5.97	-6.56
**G**	0.24	9.96	-4.66
**G.O**	0.47	6.23	-9.26

S4. pH after diluting vaginal sheets on mVFS pH 5 (1:10 w/w) after storage for 3 months. Results are presented as mean values ± standard deviation (SD), n = 3.

	**pH dilution with mVFS pH5 1:10 w/w pH + S.D (n=3) t0**	**pH dilution with mVFS pH5 1:10 w/w pH + S.D (n=3) t3 RT**	**pH dilution with mVFS pH5 1:10 w/w pH + S.D (n=3) t3 40ºC**	**pH dilution with mVFS pH5 1:10 w/w pH + S.D (n=3) t3 5ºC**
**A**	4.72 ± 0.01	4.78 ± 0.03	4.91 ± 0.02	4.79 ± 0.02
**A.O**	4.48 ± 0.02	4.62 ± 0.02	4.98 ± 0.01	4.64 ± 0.03
**B**	4.63 ± 0.03	4.69 ± 0.03	4.97 ± 0.03	4.71 ± 0.01
**B.O**	4.47 ± 0.03	4.53 ± 0.03	4.89 ± 0.02	4.60 ± 0.01
**C**	4.61 ± 0.01	4.64 ± 0.01	5.04 ± 0.02	4.69 ± 0.03
**C.O**	4.45 ± 0.01	4.51 ± 0.01	4.99 ± 0.03	4.61 ± 0.02
**D**	4.67 ± 0.01	4.71 ± 0.01	4.81 ± 0.02	4.72 ± 0.02
**D.O**	4.52 ± 0.01	4.69 ± 0.02	4.79 ± 0.03	4.72 ± 0.01
**E**	4.43 ± 0.01	4.54 ± 0.01	4.97 ± 0.01	4.64 ± 0.01
**E.O**	4.38 ± 0.01	4.49 ± 0.01	4.91 ± 0.01	4.56 ± 0.02
**F**	4.52 ± 0.01	4.61 ± 0.02	5.02 ± 0.02	4.64 ± 0.02
**F.O**	4.40 ± 0.01	4.52 ± 0.01	4.92 ± 0.03	4.58 ± 0.01
**G**	4.55 ± 0.01	4.61 ± 0.01	5.01 ± 0.01	4.65 ± 0.02
**G.O**	4.40 ± 0.02	4.58 ± 0.02	4.97 ± 0.03	4.64 ± 0.02

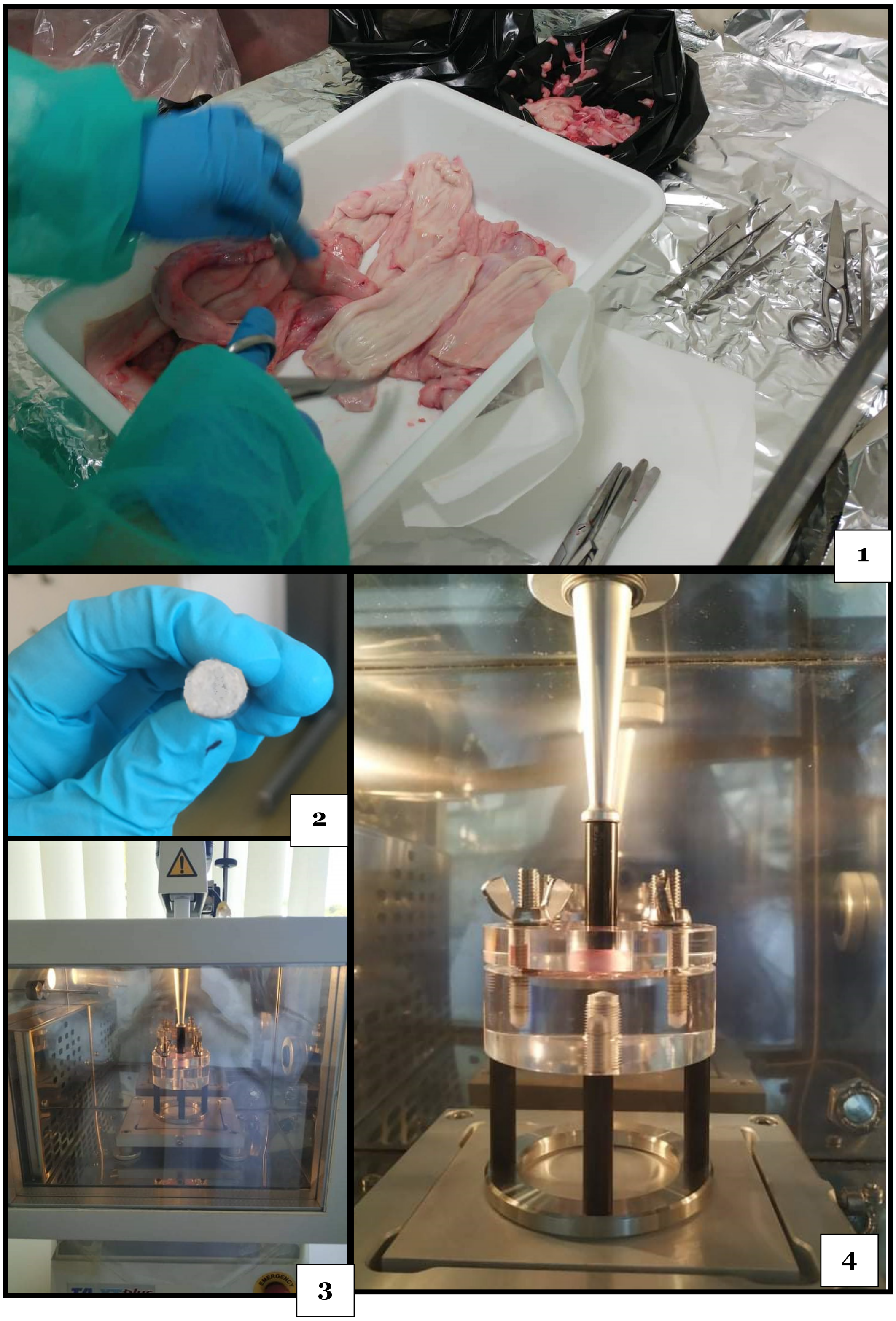

S5. Illustration of the method for evaluation of bioadhesive profile of vaginal sheets on a texturometer using *ex-vivo* porcine vaginal tissue. 1- Preparation of vaginal porcine epithelium; 2- A double sided adhesive tape allowed for circular portions of vaginal sheets attachment; 3- The whole system was maintained at 37ºC by means of an oven; 4- Porcine vaginal tissue was fixed using a mucoadhesion rig (A-MUC), avoiding its movement when the probe moves and allowing intimate contact between the formulation and the epithelium.

S6. Irritation score calculation according to the endpoint at each time point

	Score at time point		
**Endpoint**	**0.5 min**	**2 min**	**5 min**
**Lysis**	5	3	1
**Haemorrhage**	7	5	3
**Coagulation**	9	7	5
